# Comments on “Ochratoxin A: *In utero* Exposure in Mice Induces Adducts in Testicular DNA. *Toxins* 2010, *2*, 1428–1444”—Mis-Citation of Rat Literature to Justify a Hypothetical Role for Ochratoxin A in Testicular Cancer

**DOI:** 10.3390/toxins2102333

**Published:** 2010-09-29

**Authors:** Peter G. Mantle

**Affiliations:** Centre for Environmental Policy, Imperial College London, South Kensington, London, SW7 2AZ, UK; Email: p.mantle@imperial.ac.uk; Tel.: +44-207-594-5245

**Keywords:** rat testicular tumor, mouse testicular tumor, ochratoxin A, fumonisin B_1_, DNA adducts

## Abstract

A manuscript in the journal recently cited experimental rat data from two manuscripts to support plausibility of a thesis that ochratoxin A might be a cause of human testicular cancer. I believe that there is no experimental evidence that ochratoxin A produces testicular cancer in rats or mice.

In the introduction of the manuscript in question, a claim for direct evidence of a carcinogenic role for ochratoxin A (OTA) in the testis is made by citing [1], in which 25% of male rats given the toxin during the second year of life developed testis tumors. However, the experiment had no untreated animals because the limited objective was to compare duration of OTA exposure on renal carcinogenesis. Further, citation of [2] as corroborative evidence for testis carcinogenesis was even more unfortunate because it was clearly stated there that testis tumors occurred equally in treated rats and in controls. Instead, authors should have cited both the most comprehensive source of data for OTA toxicology in the rat [3], in which no evidence of additional testicular tumorigenesis attributable to the toxin was found, and the lifetime study in the mouse [4]. The latter, in which extensive histopathological study of necropsy tissues was also made, found no testis tumors in controls and none also even at the very high dietary OTA concentration of 40 ppm for up to two years. In kidney, OTA-generated tumors were abundant (50%) in the 40 ppm dietary regimen group, but only in males, and none was found in a 1 ppm regimen group.

Thus, the literature clearly states that OTA did not cause testicular tumors in rats, or in mice even when high dose exposure severely constrained growth [3] and provided approximately six times more daily OTA exposure than in [5] without causing testis tumors. Further support comes indirectly from a more recent NTP study on fumonisin B_1_ [6], in which testicular cancer was naturally common in ageing control male rats and similarly so in response to increasing exposure to that toxin. Also, there were no testis tumors in male mice. Consequently, at the end of the discussion in [5], it is incorrect to use the findings to explain origin of the natural testis tumors described in [1] and [2].

Consequently, the unfortunate mis-citation of [1] and [2] leaves a role for OTA in human testicular cancer unsupported by model animal experiments and still needing DNA adduct data to show whether there are any vascularized tissues in which adducts are not found. A simple pattern of one or two rat kidney adducts has been shown by ^32^P post-labeling in response to OTA [7]. The dominant one has been characterized as a covalent DNA adduct between the des-chloro-analogue of OTA and guanine, and its potential to cause genetic change via subsequent mis-repair has been recognized [7]. However, the significance of greater complexity of some adduct patterns in [5] remains to be elucidated. The justification for a hypothesis that OTA might cause testicular cancer [5] includes reference to Balkan endemic nephropathy (BEN), and “high levels of OTA-contamination of food” in the Balkans. However, increased incidence of testicular cancer has not been recognized as associated with the bilateral renal atrophy of BEN nor of the transitional cell carcinomas in the upper urinary tract which are sometimes associated with BEN. If exceptional significant exposure to OTA is associated with either the renal atrophy or the urinary tract tumors, there should have been a concomitant increase in testicular cancer if human testes have a propensity to respond adversely to circulating OTA. Also, a difference of <1 µg in the mean daily individual intake of OTA, between BEN and non-BEN households [8], can hardly justify “high level” as a descriptor of BEN household intake. Further, on a body weight basis, mean daily OTA intake (1.2 µg) in BEN households [8] was ~500 times less than that causing just 2 adducts/10^9^ nucleotides in mouse testis [5]. It is therefore very difficult to see experimental evidence supporting the original hypothesis which suggested that OTA could be a cause of human testicular cancer [9]. 

Rat seminomas associated with exposure to OTA have not previously been illustrated, probably because no etiological significance of the toxin was attributed in the NTP study [5]. Figure 1 shows testis histopathology in a two-year-old rat, euthanized prematurely because of a large, age-related, subcutaneous tumor after 90 weeks of exposure to OTA at twice the mean daily rate of the highest dose in the NTP study [5]. A steady plasma OTA concentration of ~11.4 µg/mL was achieved [10] and the cumulative OTA dose was >70 mg. Bi-lateral renal tumors, typically attributed to the dietary OTA exposure regimen, also occurred, and confirmed the carcinogenicity of OTA in this animal. There was no confounding clinical or histopathological evidence of the mononuclear leukemia to which F 344 rats are prone. This testis tissue, and that of others in my archive of tumorous testis tissue of defined experimental OTA provenance, can be available for further study if requested.

**Figure 1:**
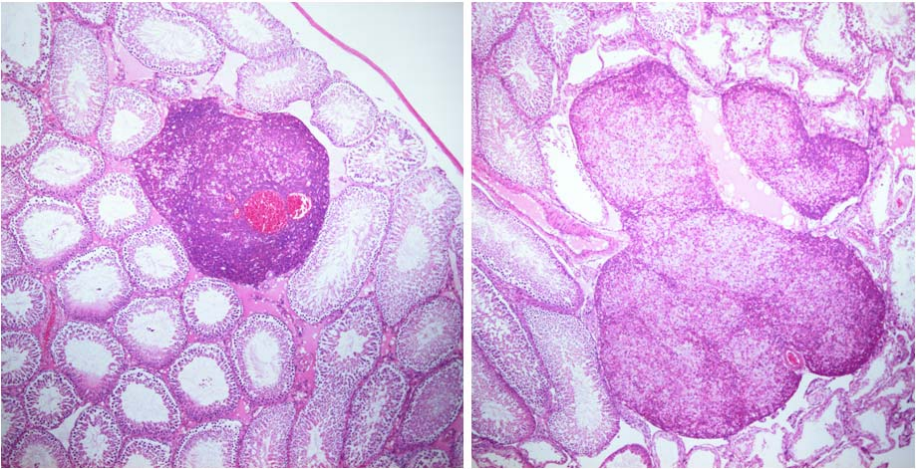
Two regions of a testis of an F 344 rat, which ingested OTA (300 µg/kg body weight) daily in feed for 90 weeks, in a longitudinal section (1.0 × 1.8 cm) stained with hematoxylin and eosin showing vascularized seminomas amongst ageing seminiferous tubules. A causal association is not implied for the toxin because testicular tumor is natural in some ageing rats.

## References

[1] Mantle P.G., Nolan C.C. (2010). Pathological outcomes in kidney and brain of male Fischer rats given dietary ochratoxin A, commencing at one year of age. Toxins.

[2] Mantle P., Kulinskaya E., Nestler S. (2005). Renal tumourigenesis in male rats in response to chronic dietary ochratoxin A. Food Addit. Contam..

[3] Boorman G.A. (1989). Toxicology and Carcinogenesis Studies of Ochratoxin A (CAS No. 303-47-9) in F344/N Rats (Gavage Studies); National Toxicology Program Technical Report No. 358.

[4] Bendele A.M., Carlton W.W., Krogh P., Lillehoj E.B. (1985). Ochratoxin A carcinogenesis in the (C57BL/6J x C3H)F1 mouse. J. Natl. Cancer Inst..

[5] Jennings-Gee J.E., Tozlovanu M., Manderville R., Miller M.S., Pfohl-Leszkowicz A., Schwartz G.G. (2010). Ochratoxin A: *In utero* exposure in mice induces adducts in testicular DNA. Toxins.

[6] Toxicology and carcinogenesis studies of fumonisin B1 in F344/N rats and B6C3F1 mice (feed studies). NTP Technical Report No. 496. http://ntp.niehs.nih.gov/ntp/htdocs/LT_rpts/tr496.pdf.

[7] Mantle P.G., Faucet-Marquis V., Manderville R.A., Squillaci B., Pfohl-Leszkowicz A. (2010). Structures of covalent adducts between DNA and ochratoxin A: a new factor in debate about genotoxicity and human risk assessment. Chem. Res. Toxicol..

[8] Abouzied M.M., Horvath A.D., Podlesny P.M., Regina N.P., Metodiev V.D., Kamenova-Tozeva R.M., Niagolova N.D., Stein A.D., Petropoulos E.A., Ganev V.S. (2002). Ochratoxin A concentrations in food and feed from a region with Balkan Endemic Nephropathy. Food Addit. Contam..

[9] Schwartz G.G. (2002). Hypothesis: Does ochratoxin A cause testicular cancer?. Cancer Causes Control.

[10] Mantle P.G. (2008). Interpretation of pharmacodynamics of ochratoxin A in blood plasma of rats, during and after acute or chronic ingestion. Food Chem. Toxicol..

